# Systemic Lisbon Battery: Normative Data for Memory and Attention Assessments

**DOI:** 10.2196/rehab.4155

**Published:** 2016-05-04

**Authors:** Pedro Gamito, Diogo Morais, Jorge Oliveira, Paulo Ferreira Lopes, Luís Felipe Picareli, Marcelo Matias, Sara Correia, Rodrigo Brito

**Affiliations:** ^1^Cognition and People-centric Computing Laboratories– COPELABS. Lusophone University of Humanities and TechnologiesLisbonPortugal; ^2^School of Psychology and Life Sciences. Lusophone University of Humanities and Techologies.LisbonPortugal

**Keywords:** Systemic Lisbon Battery, attention, memory, cognitive assessment, virtual reality

## Abstract

**Background:**

Memory and attention are two cognitive domains pivotal for the performance of instrumental activities of daily living (IADLs). The assessment of these functions is still widely carried out with pencil-and-paper tests, which lack ecological validity. The evaluation of cognitive and memory functions while the patients are performing IADLs should contribute to the ecological validity of the evaluation process.

**Objective:**

The objective of this study is to establish normative data from virtual reality (VR) IADLs designed to activate memory and attention functions.

**Methods:**

A total of 243 non-clinical participants carried out a paper-and-pencil Mini-Mental State Examination (MMSE) and performed 3 VR activities: art gallery visual matching task, supermarket shopping task, and memory fruit matching game. The data (execution time and errors, and money spent in the case of the supermarket activity) was automatically generated from the app.

**Results:**

Outcomes were computed using non-parametric statistics, due to non-normality of distributions. Age, academic qualifications, and computer experience all had significant effects on most measures. Normative values for different levels of these measures were defined.

**Conclusions:**

Age, academic qualifications, and computer experience should be taken into account while using our VR-based platform for cognitive assessment purposes.

## Introduction

Attention and memory are among the most common cognitive functions affected by acquired brain injuries [1]. Attention refers to the process of selecting a specific stimulus from the physical environment (external stimuli) or the body (internal stimuli) [2]. In addition to selection, this ability also depends on processes of orientation and alertness [3]. The symptoms resulting from the disruption of these abilities are related to an inability to process information automatically. Tasks that are usually automatic (eg, reading) become more difficult for patients with brain injuries, and require a great deal of effort and concentration [4,5]. The neural basis for attention may rely on different brain areas, from midbrain structures [6], to parietal regions, and the anterior pre-frontal cortex [7,8]. The ability to perform everyday life tasks may also depend on memory functions [9], which are particularly affected by pre-frontal brain lesions [10]. Memory can be defined as the ability to encode and/or recall a specific stimulus or situation. There are different theoretical and clinical models that conceptualize memory in terms of information (declarative vs non-declarative) or temporal dimensions (retrospective vs prospective). One model of memory suggests, for example, that information can be manipulated in memory before it is used for a specific purpose [11]. This ability has been defined as working memory, which consists of multiple subsystems that store (for a limited amount of time in short-term memory), and manipulate different kinds of sensory information [12]. However, the roles of attention and memory abilities in everyday functioning go beyond these specific processes, being related to a wider range of cognitive functions called executive functions [13].

The ability to prepare a meal accurately by being able to maintain an adequate level of attention to the task, or even remembering what to buy at the grocery shop, are examples of attention or memory abilities applied to different domains of instrumental activities of daily living (IADLs) that are usually compromised, to different extents, by traumatic brain injuries [14], stroke [15], or even alcohol abuse [16].

The assessment of attention and memory functions is traditionally made with paper-and-pencil tests. Cancelation tests for visual stimuli are usually the best option to assess attention abilities, whilst the Wechsler Memory Scale is one of the most widely used tests for memory assessment [17,18]. This test assesses memory functions within different domains, comprising the following seven subtests: (1) spatial addition, (2) symbol span, (3) design memory, (4) general cognitive screener, (5) logical memory, (6)verbal paired associates, and (7) visual reproduction. In addition to the partial scores on each subtest, total scores reflect general memory ability. One of the shortcomings of such tests, however, is the fact that they do not evaluate the patient while he or she is performing IADLs. Their ecological validity is, therefore, uncertain [19-21]. The optimal way to avoid this pitfall is to carry out evaluations of cognitive performance based on IADLs. While pervasive technologies are already available to contribute to this purpose through the collection of behavioral and physiological data [22], the correlation between the collected data and the impairment of a specific domain, such as memory and attention, has not yet been established.

An emerging alternative to traditional tests is to design and develop virtual reality (VR) worlds that mimic real IADLs and record participants’ performance while executing specific tasks involving attention and memory functions. One such platform is the basis for the Systemic Lisbon Battery (SLB) [23]. It consists of a 3D mock-up of a small town in which participants are free to walk around and engage in several IADLs and in ordinary digital games. While these activities are taking place, the system records for each task several indicators of performance, such as errors and execution times. In order for this to fulfill its purpose of assessment, it must be ensured that the SLB activities are valid indicators of functionality for the cognitive dimension that they were designed to assess. For the virtual kitchen, one of the activities of the SLB, this has already been established [24] using the Virtual Kitchen Test (VKT). The VKT was designed to evaluate frontal brain functioning and was pre-validated in a controlled study with a clinical sample of individuals with alcohol dependence syndrome and with cognitive impairments. This test was developed according to the rationale of the Trail Making Test [25], which is a well-established test used to assess frontal functions. The results showed that scores from the VKT were associated with participants’ performance on traditional neuropsychological tests, and discriminated between the cognitive performance of patients and controls involved in the study.

Another recent study has focused on defining normative data based on which clinical deviations could be identified for each IADL activity and/or task in the SLB [23]. In that study, 59 healthy students performed the exercises available in the SLB that address attention and memory functions. The results of that study suggested that this approach may be an alternative to traditional neuropsychological tests, but broader samples were needed to establish the normative values of performance in those tests with greater confidence. Here, our aim was to estimate normative scores for the SLB from a larger, non-clinical sample collected in the general population, as well as to test the concurrent validity of the SLB subscales with conventional neuropsychological tests.

## Methods

### Participants

We used a snowball method for recruiting participants. Masters students enrolled in a course on cyberpsychology were specially trained for this study and recruited family members (ie, siblings, parents, and grandparents) to participate. This ensured some demographic diversity through the participation of roughly three different cohorts of adults of both genders. These were asked to participate in a study designed to evaluate attention and memory performance while executing VR-based daily life activities. Participants were not included if they were younger than 18 years of age, had a history of psychiatric disorders, perceptual or motor disabilities or substance abuse. In addition, participants were excluded if they did not have regular access to the World Wide Web and/or if they scored below the cutoff values for their age on the Mini-Mental State Examination (MMSE) [26], which was administered prior to the main tasks. However, all participants scored above those cutoff points.

A final sample of 243 participants with a mean age of 37 years (SD 15.87), 39.5% male (96/243), and 60.5% female (147/243), was included in the study. Of the participants, 69.5% (169/243) had previous experience in using a personal computer for gaming purposes. Formal education ranged from 9 years to post-graduate level, with completed secondary-level studies (27.2%, 66/243) and ongoing university studies (23.0%, 55/243) the most frequent responses. A characterization of the participant sample is detailed in Table 1.

**Table 1:** Sample characterization (N=243).

Characterization		n (%)
Gender		
	Male	96 (39.5)
	Female	147 (60.5)
Employment situation		
	Student	71 (30.1)
	Working student	1 (0.4)
	Worker	144 (61.0)
	Unemployed	9 (3.8)
	Retired	11 (4.7)
Computer experience		
	None	22 (9.1)
	Basic	88 (36.2)
	Intermediate	116 (47.7)
	Expert	17 (7.0)
Video game experience		
	Never	107 (44.8)
	Occasionally	88 (36.8)
	Frequently	30 (12.6)
	More than 50% of days	9 (3.8)
	Every day	5 (2.1)
Formal education		
	Basic studies	43 (18.0)
	Incomplete high school	32 (13.4)
	High school	65 (27.2)
	University studies	55 (23.0)
	University degree	35 (14.6)
	Graduate Studies	9 (3.8)
Age, years		
	Mean (SD)	36.99 (15.85)
	Minimum	18
	Maximum	86
MMSE score		
	Mean (SD)	28.09 (3.09)
	Minimum	22
	Maximum	30

### Study Procedure

Potential participants first responded to a screening protocol questionnaire. If they did not fulfill all the inclusion criteria, they were thanked and did not participate in the study. Participants fulfilling the inclusion criteria were given the MMSE test, but their results on the MMSE were only analyzed after their participation in the main task. Both the MMSE and the screening protocol used to assess the other criteria were administered in paper forms. Interviewers then ran Unity Web Player and asked participants to sign in to the platform with a pre-established code so that we could, if needed, establish an epigenetic relation between participants. Before performing the main tasks, participants carried out a familiarization task to ensure that they had the necessary skills to navigate and interact in a mediated 3D environment, but this task did not include the tasks on which they would be assessed.

The main tasks were carried out on the SLB [23], a VR platform for the assessment of cognitive impairments based on serious-games principles and developed on Unity 2.5. It consists of a small-city scenario, complete with streets, buildings, and normal infrastructures (eg, shops) used by people in their daily lives. The SLB is freely available online [27]. To ensure a more immersive environment, tthe SLB scenario is populated by computer-controlled non-playable characters (NPCs), which roam across the city. Besides the house, which is the spawn point (the starting point of the player in scenario), and in which the users can engage in most of the home-based daily activities (ie, personal hygiene, dressing, meals), this "city" has a supermarket, an art gallery, a pharmacy, and a casino. The assessment tasks are performed in all these settings. The tasks to perform in the SLB range from memory tasks to complex procedures, and the platform is undergoing a constant process of development to optimize and expand the set of tasks included.

**Figure 1:**
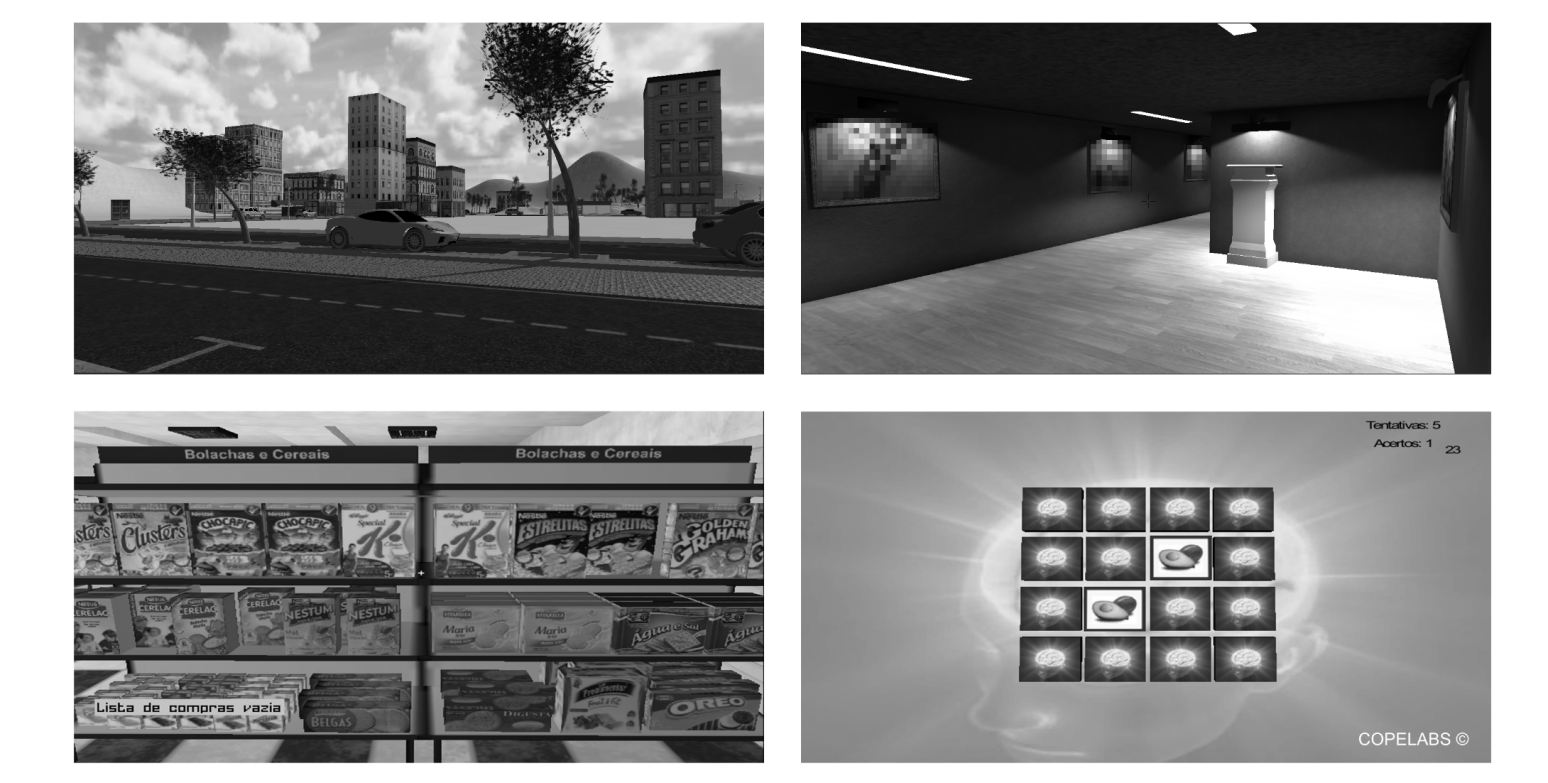
Systemic Lisbon Battery (SLB) subtests. City spawn point (top left); gallery (top right); supermarket (bottom left); and memory game (bottom right).

In this particular study, participants performed three different tasks. The first (fruit-matching) is a short-term memory task consisting of a matching tiles game in which participants had to complete 8 trials of matching pairs of fruits. The second (supermarket) is a working memory and attention task, and took place in a supermarket scenario where the participants were instructed to buy 7 products (a milk bottle, a pack of sugar, a bottle of olive oil, a pack of crackers, a bottle of soda, a bottle of beer, and a can of tuna) for the lowest possible expense (€25 maximum) in a minimum amount of time. The third (art gallery) is an attention task, and took place in an art gallery. Participants had to match missing pieces in three different paintings into their correct place. These three tasks are illustrated in Figure 1.

The avatar was spawned in the bedroom, where the participant had to complete the first task. The other tasks were performed according to a protocol that was provided on screen just before signing in. All activities were listed in the protocol, together with the indications to roam the virtual city. For each task, performance indicators were automatically recorded, for each code, in a file (*.txt) that was later exported to Microsoft Excel.

### Outcome Measures and Statistical Analysis

Basic cognitive performance was assessed with the MMSE [26] in a validated Portuguese version [28,29]. The MMSE is a brief screening test that assesses aspects of mental function related to cognition. Higher scores on each measure indicate better cognitive functionality. We used the cutoff values for the Portuguese population established by Guerreiro and colleagues [28], according to level of education: 22 for 0-2 years of schooling, 24 for 3-6 years of schooling and 27 for 7 or more years of schooling.

IADLs-related cognitive performance measures were based on the execution times and number of errors in the three SLB tasks (fruit matching memory task, supermarket memory and attention task, and art gallery attention task). We verified the correlations between these to avoid measurement overlap. In the case of the supermarket task, in which participants were instructed to go for the cheapest solutions, we also added the amount of cash spent on listed products. In all cases, lower scores indicate higher cognitive performance.

The main goal of this study was to establish normative values for three subtests of the SLB. Given what is known on the effect of demographic variables (ie, namely age and education) on measures of cognitive performance, it was important to identify their effects and establish normative values separately for different levels of age and education. Since the SLB was developed as a VR application, controlling the effects of video game and computer experience was also a necessary goal. Finally, we were also interested in understanding the relations between the results of the different subtests, as well as between the different subtests results and MMSE.

Demographic effects on performance were tested with non-parametric statistical analyses (Mann-Whitney and Kruskal-Wallis tests for independent samples), since the distributions of the performance measures did not pass the Kolmogorov-Smirnov test for normality. For the same reason, we computed correlations using Spearman's rank order correlation (ρ).

Inferential statistics were carried out using IBM SPSS v.20 (IBM Corp. USA). For every statistical analysis, we considered a CI of 95%, so statistical results are reported as significant when the *P* value is lower than .05. Although this was not an experimental study, the main conclusions of this study were based on inferential statistics, which required a priori power analysis to estimate the sample size needed for proper statistical analysis. This procedure was conducted in G*Power (v3.1) with Cohen’s *r* effect size for non-parametric Spearman’s *r*_*s*_tests [30,31]. Given an expected effect size of .30 (medium) for a .05 significance level (alpha) in two-tailed testing with a power (1-beta) of .80, the required sample size for this study was 167 participants.

## Results

Means (SDs) for both errors and execution time for the three subtests are reported in Table 2. For subtest 2 (supermarket), the descriptive statistics for money spent are also reported. Finally, CIs for the 95% level are also provided for each subtest. The correlations between execution times on the three different tests were all positive and moderate: gallery–memory game, *r*_*s*_(128) = .371, *P*<.001; gallery-supermarket, *r*_*s*_(127) = .312, *P*=.001; memory game – supermarket, *r*_*s*_(116) = .360, *P*<.001, suggesting that time-performance on the different SLB tasks is evaluating interrelated cognitive performance constructs. The inter-correlations between execution times and errors *within* each subtest were also all positive and moderate: gallery, *r*_*s*_(125) = .300, *P*=.001; memory game, *r*_*s*_(103) = .341, *P*<.001: and supermarket, *r*_*s*_(129) = .510, *P*<.001; which is what we should expect. However, none of the correlations between error rates in the different subtests were significant, which is a result that needs some discussion. In addition, the predictably negative correlations with task scores on the MMSE were all either weak or non-significant.

**Table 2:** Descriptive data on virtual reality-based subtests (N=243).

	Mean (SD)	CI 95%	
		Lower bound	Upper bound
Memory game execution time	40.66 (8.94)	37.29	40.91
Memory game errors^a^	7.85 (2.47)	7.43	8.49
Supermarket execution time	435.98 (202.37)	394.31	477.66
Supermarket errors	7.19 (9.59)	5.22	9.17
Supermarket money spent^b^	10.56 (3.88)	9.76	11.36
Gallery execution time	155.55 (105.12)	119.79	155.20
Gallery errors	10.64 (18.47)	5.94	13.39

^a^Number of incorrect hits.

^b^Money spent in Euros used to purchase the pre-defined list of products.

In order to test whether there were effects of socio-demographic characteristics and computer and video game experience on performance in this set of subtests of the SLB, and thus if separate normative values should be established for different levels of each of those variables, we carried out a series of tests. Since most of the outcome variables were non-normally distributed, we used either the Mann-Whitney or the Kruskal-Wallis tests, respectively, for two or more groups.

The test for computer experience (Table 3) indicates effects on execution times in both the fruit matching memory task (χ^2^_3_=12.485, *P*=.006), and in the art gallery attention task (χ^2^_3_=9.351, *P*=.025). In the memory task, specialists performed significantly faster than participants with no experience (*P*=.008), basic experience (*P*=.001), and intermediate experience (*P*=.012). In the art gallery attention task, specialists also performed significantly faster than participants with no experience (*P*=.012), with basic experience (*P*=.006), and with intermediate experience (*P*=.036). In fact, participants with a lot of computer experience were typically much faster than other participants in performing the tasks, suggesting that computer experience should be taken into account when assessing performance based on execution times.

As for academic qualifications, tests results show one significant effect on performance as measured by number of errors in the art gallery attention task (χ^2^_5_= 22.024, *P*=.001). Here, the significant differences were between participants with only basic studies, on the one hand, and on the other, those who had completed high-school (*P*=.000), had or were attending university (*P*=.013), or had university degrees (*P*=.006) (Table 4). This task thus seems to be tapping into some cognitive skill that is learned in the high school system. There were no significant effects of gender, video game experience, TV viewing-hours per week, VR knowledge, 3D experience, or 3D knowledge, on any of the cognitive performance indicators.

**Table 3:** Subtests results by computer experience.

	Level of computer experience, mean ranks				
	None	Basic	Intermediate	Specialist	χ^2a^
Memory game execution time	72.68	74.96	61.98	24.00	12.485^b^
Memory game errors^c^	38.00	52.08	60.78	45.89	5.581
Supermarket execution time	84.40	68.25	60.40	54.50	4.619
Supermarket errors	75.40	66.30	60.87	74.69	2.132
Supermarket money spent^d^	81.75	61.91	63.53	74.75	3.067
Gallery execution time	75.90	71.31	60.85	29.86	9.351^e^
Gallery errors	82.70	62.49	61.86	45.67	4.476

^a^Chi-square of the Kruskal-Wallis test.

^b^ *P*<.01.

^c^Number of incorrect hits.

^d^Money spent in Euros used to purchase the pre-defined list of products.

^e^ *P*<.05.

**Table 4:** Subtests results by academic qualifications.

	Level of academic qualification, mean ranks						
	Basic studies (9^th^grade)	Incomplete high school	High school	University attendance	University degree	Graduate studies	χ^2a^
Memory game execution time	67.23	75.33	67.09	59.04	57.65	44.00	4.774
Memory game errors^b^	42.36	64.97	60.54	50.39	51.41	42.17	7.694
Supermarket execution time	76.54	55.82	57.88	55.09	76.90	50.33	9.731
Supermarket errors	76.48	56.94	59.97	57.61	64.62	69.58	5.130
Supermarket money spent^c^	77.71	67.44	59.97	55.13	65.29	41.25	8.269
Gallery execution time	67.32	68.33	59.21	60.95	58.06	69.83	1.761
Gallery errors	84.00	73.88	46.35	54.00	54.53	52.00	22.024^d^

^a^Chi-square of the Kruskal-Wallis test.

^b^Number of incorrect hits.

^c^Money spent in Euros used to purchase the pre-defined list of products.

^d^ *P*<.01.

Age was significantly, albeit only weakly or at best moderately, related to reduced performance, as measured by execution times on the different tasks: art gallery attention task *r*_*s*_(127) = .312, *P*<.001; fruit matching memory task *r*_*s*_(139) = .172, *P*=.049; supermarket memory and attention task *r*_*s*_(127) = .184, *P*<.001), as well as by the MMSE *r*_*s*_(242) = -.147, *P*=.022. We tested the effects of age cohort on performance in the different subtests of the SLB (execution time and errors for both gallery and memory game) by dividing the sample into four cohorts according to age quartiles (Table 5). Results indicate significant effects for both gallery execution time (χ^2^_3_=14.733, *P*=.002) and gallery errors (χ^2^_3_=10.400, *P*=.015). Older participants took longer to complete the task and made more errors. Post-hoc comparisons show significant differences in the gallery execution time measure in the comparisons between the <23 years age group and both the >49 and 35-48 age groups. With respect to gallery errors, the most significant differences were between the 23 to 34 and the >49 age groups. The age effect for the memory task (execution time) was just beyond the margin of significance (*P*=.052), so we did not analyze post-hoc differences.

**Table 5:** Kruskal-Wallis non-parametric comparison of Systemic Lisbon Battery (SLB) performance by age cohorts.

	Age cohort in years, mean (SD)				χ^2^
	<23	23-34	35-48	>49	
Gallery execution time	105.31 (57.36)	135.98 (69.96)	173.70 (130.58)	186.08 (103.22)	14.733^a^
Gallery errors^b^	5.69 (9.20)	3.22 (3.93)	9.91 (16.50)	19.46 (26.45)	10.400^c^
Memory game execution time	36.85 (9.19)	43.01 (7.43)	42.45 (8.19)	40.58 (9.51)	7.721
Memory game errors	8.21 (2.49)	7.86 (2.11)	8.061 (2.46)	7.33 (2.62)	.544

^a^ *P*<.01.

^b^Number of incorrect hits.

^c^ *P*<.05.

## Discussion

### Principal Findings

Neuropsychological research has exposed the limitations of traditional paper-and-pencil neuropsychological tests for the assessment of cognitive functioning. A major critique is that those tests do not replicate cognitive functions used in the activities of daily living. A more ecologically valid emerging alternative is to use VR-based applications to test executive functions and related cognitive functions such as memory and attention. One of these applications is the SLB [23], a free online application and cognitive test, which provides a highly immersive and motivating experience with a first-person view that mimics IADLs.

The main objective of this study was to identify normative values for this application to be used as baseline in clinical studies. Our results indicate that performance on VR-based IADLs as measured by execution times is enhanced by education and computer experience, whilst age decreases performance. According to these results, we propose that normative values for execution times on VR-based IADLs be separately established for different levels of each of these variables. Conversely, we found no effects of gender, which is reassuring in that it indicates that the SLB has no gender bias and normative values do not need to be adjusted to gender. In addition, the moderate positive correlations between execution times suggest that the different subtests are tapping into different but associated cognitive functions, which is what we would have expected. The same pattern was not found for errors, which is probably due to floor effects on all of these, as we are dealing with a non-clinical sample for which errors are all relatively low. However, error rates on each of the tasks are correlated with the respective execution times, which indicate they are not random. Correlations between task performance and MMSE are mostly non-significant, which is probably due to a ceiling effect on the MMSE itself, also typical of non-clinical samples.

If we take into account these differences, these results indicate that VR-based assessments of cognitive functions using tasks that reproduce activities of daily life, such as the SLB, may be useful to assess cognitive functioning during the execution of activities of daily living, although a larger study comparing normal with clinical samples, and evaluating the comparative performance and within-subject correlation between results of the SLB and traditional neuropsychological tests is still needed. Moreover, it is important to note that it is possible to have these applications available anytime, anywhere, and to everyone with the advent of pervasive technology through mobile devices, which will make their use easier and more accessible than current conventional treatments. It is therefore urgent to test their validity and establish normative data for varied populations.

### Limitations

The data was recorded on a variety of personal computers and several volunteers participated in the data collection. Thus, it was impossible to guarantee homogeneity of conditions, in particular in what concerns the influence of screen types, interfaces (eg, mouse), and of the person running the tests. The over-65-years sample size was too small to draw firm conclusions for that age group. In addition, we did not assess whether prior training could impact on performance, as all participants underwent training before the assessment. Finally, the fact that we were assessing a non-clinical sample probably explains the floor and ceiling effects in the error rates and MMSE, even though this is an essential step for every assessment and/or training battery validation that is focused in working with both clinical and general populations.

### Conclusions

The assessment of cognitive functions is traditionally made through non-ecological pencil-and-paper tests. However, interactive and immersive platforms options like virtual reality apps, which mimic real-life activities, are increasingly available. Nevertheless, such options require establishing normative data for healthy populations, which can be used to assess cognitive problems in (potentially) clinical populations. This study follows this aim by identifying normal levels of cognitive performance in a non-clinical sample, using assessment measures based on VR versions of IADLs chosen for their demand on memory and attention functions. Age, level of education, and computer experience all appear to contribute to performance with this tool, which implies that normative values have to be adjusted to all these variables.

## Abbreviations

IADLs: instrumental activities of daily living
MMSE: Mini-Mental State Examination
SLB: Systemic Lisbon Battery
VKT: Virtual Kitchen Test
VR: virtual reality
